# An Innovative Technique For Posterior Inferior Cerebellar Artery (PICA) Transposition During Foramen Magnum Meningioma Resection: A Case Report

**DOI:** 10.7759/cureus.89058

**Published:** 2025-07-30

**Authors:** Eddie Lim, Davendran Kanesen

**Affiliations:** 1 Neurosurgery, Sarawak General Hospital, Kuching, MYS

**Keywords:** foramen magnum meningioma, skull base meningioma, suboccipital craniotomy, transposition, vascular preservation in neurosurgery

## Abstract

This report outlines the successful excision of an anterolateral foramen magnum meningioma (FMM) in a 69-year-old female patient who exhibited symptoms of vertigo, numbness in the left shoulder, and a fall. Neuroimaging revealed a lesion located at the anterolateral foramen magnum. The tumor was removed using a posterior midline suboccipital approach, with meticulous dissection performed around the left posterior inferior cerebellar artery (PICA). This artery was transposed with the aid of a Yasargil 5 mm curved temporary fenestrated clip (Aesculap AG & Co., Tuttlingen, Germany) to enable a Simpson grade 2 resection. The postoperative recovery was smooth, resulting in discharge on the third day, and histopathological analysis confirmed the diagnosis of an angiomatous meningioma, classified as CNS WHO grade 1. This case highlights the practicality and efficacy of PICA transposition during the resection of anterolateral FMM, showcasing the innovative application of a temporary fenestrated clip to stabilize the vessel while maintaining vascular integrity, thus allowing for safe tumor removal. This technique presents a promising approach for the management of complex foramen magnum lesions, yielding favorable results.

## Introduction

The foramen magnum, an important aperture situated at the lower part of the occipital bone, functions as the exit pathway for the spinal cord. Foramen magnum meningioma (FMM) accounts for approximately 0.3%-3.2% of all meningiomas and presents significant surgical challenges owing to its close association with vital neurovascular elements, such as the lower cranial nerves, vertebral arteries, and the posterior inferior cerebellar artery (PICA) [1]. The primary objective of treatment is the complete resection of these tumors, and the Simpson grading system is commonly employed to assess the degree of surgical resection and to forecast the likelihood of recurrence [2]. However, achieving a Simpson grade 1 or 2 resection in ventrolateral FMMs presents significant challenges, primarily due to their anatomical positioning and the likelihood of encasing surrounding neurovascular structures [3]. Effective surgical planning is crucial for identifying the optimal technique and reducing the likelihood of complications. The far lateral approach has been extensively regarded as the preferred method for the excision of intradural ventral and ventrolateral FMMs [4,5]. Transposing the PICA, which frequently runs along the tumor's surface, is a crucial step that enhances access to the lesion while maintaining the integrity of the vascular structures. This report details a technical case that successfully involved the resection of a ventrolateral FMM, utilizing PICA transposition achieved through the application of a Yasargil 5 mm curved temporary fenestrated clip (Aesculap AG & Co., Tuttlingen, Germany).

## Case presentation

**Figure 1:**
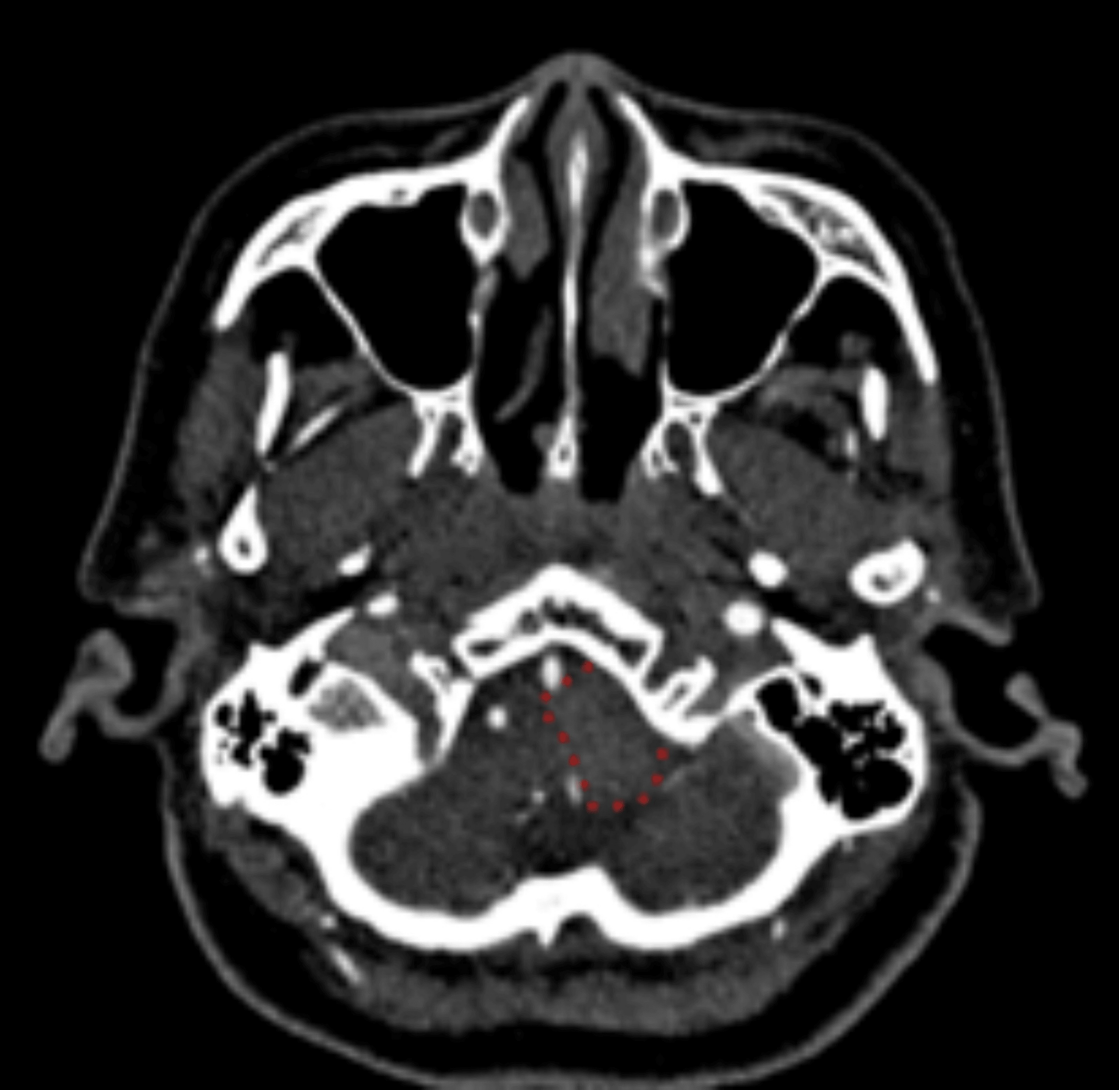
Contrast-enhanced CT of the brain showing an isodense lesion with minimal contrast uptake in the foramen magnum, adjacent to the left vertebral artery. The lesion boundary is outlined with red dots.

A 69-year-old Chinese woman with essential hypertension presented with a sudden fall, vertigo, and left shoulder numbness, without loss of consciousness or systemic symptoms. Clinical examination was unremarkable, showing no neurological deficits or cerebellar dysfunction. Cardiovascular assessments, including cardiac markers, ECG, and echocardiogram, were normal. Given the unique presentation of a drop attack, CT brain imaging revealed an ill-defined, minimally contrast-enhancing extra-axial lesion at the foramen magnum adjacent to the left vertebral artery (VA) (Figure 1).

**Figure 2:**
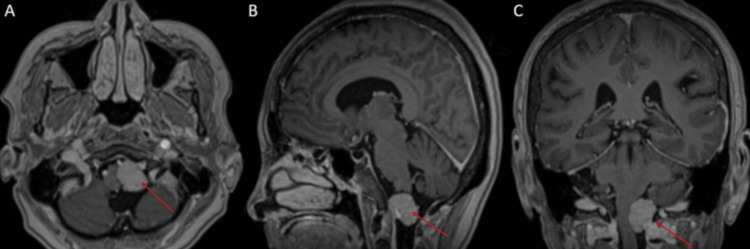
(A) Axial, (B) sagittal, and (C) coronal T1-weighted MRI with gadolinium enhancement revealing a well-defined, homogeneously enhancing mass (red arrow) at the left foramen magnum, measuring 21 x 20 x 23 mm. The mass exerts pressure on the left medulla, causing rightward deviation.

**Figure 3:**
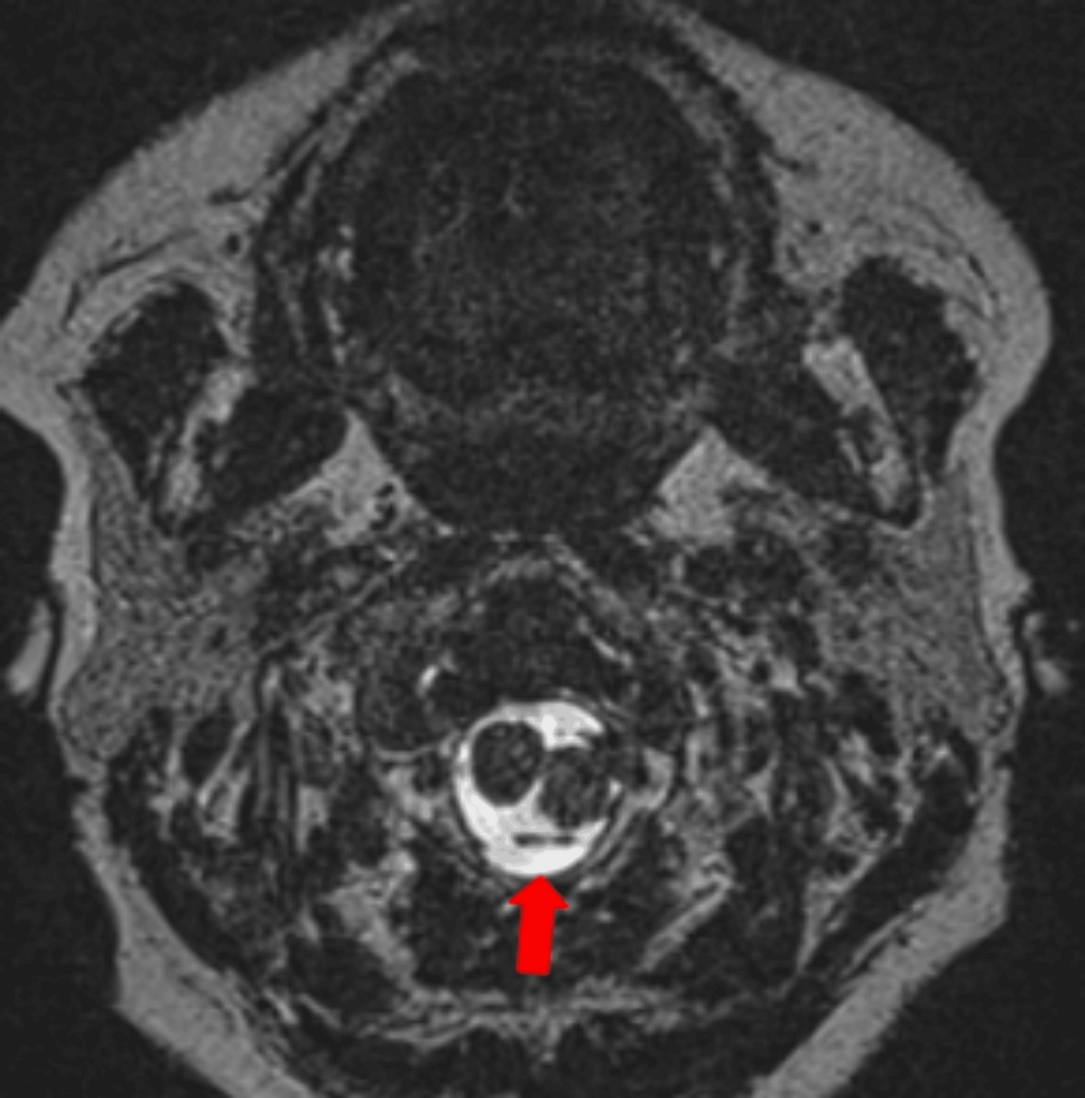
MR angiography revealing the left PICA (red arrow), located posterior to the mass. PICA: posterior inferior cerebellar artery.

MRI (Figure 2) and MR angiography (Figure 3) confirmed a homogeneously enhancing extra-axial lesion on the left side of the foramen magnum, closely associated with the V4 segment of the left VA and the left PICA.

Surgical technique

A suboccipital craniotomy and tumor excision via a midline approach were performed. Under general anesthesia, the patient was positioned prone, with the head secured in a head clamp, and the neck flexed to a thyromental distance of two fingerbreadths to optimize the cervicomedullary angle. Care was taken to avoid excessive neck flexion to prevent airway pressure elevation, venous return disruption, and pressure on the spinal cord and brainstem. Vital signs were monitored before surgery commenced.

A midline incision was made from 2 cm above the inion to the C2 spinous process. The muscles were dissected along avascular planes and detached subperiosteally from the occipital bone. Suboccipital craniotomy was performed with burr holes near the transverse sinuses and the foramen magnum, connected using a high-speed drill. Bone removal extended to the posterior edge of the foramen magnum, allowing improved access to the fourth ventricle. A C1 laminectomy was also performed bilaterally, 1 cm from the midline.

**Figure 4:**
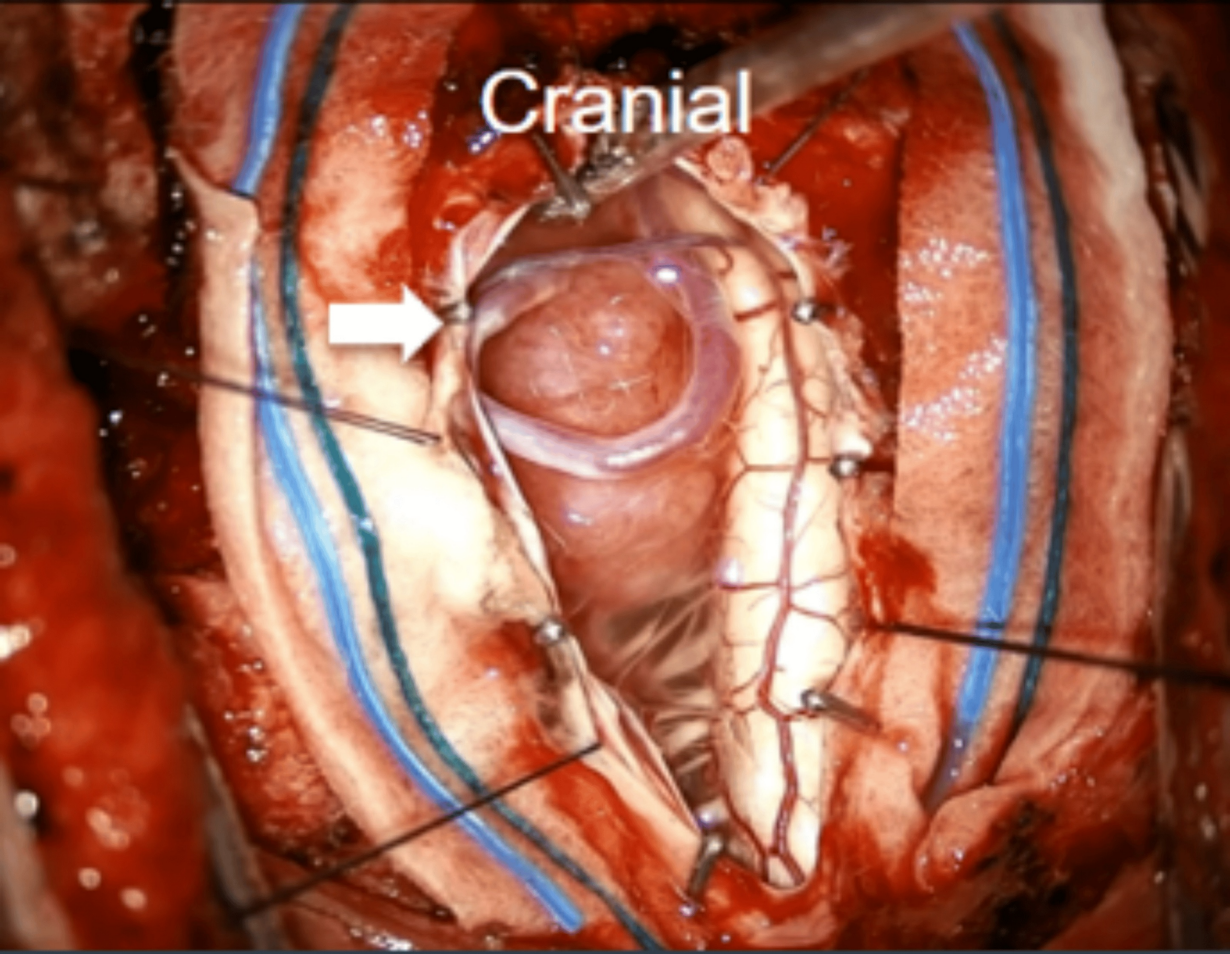
Following dura opening, a tumor was seen next to the medulla and spinal cord. The left PICA (white arrow) is also seen overlying the tumor. PICA: posterior inferior cerebellar artery.

Under a microscope, the dura was opened with a linear incision, and the arachnoid over the cerebellomedullary cistern was opened to drain CSF. The tumor, located on the left lateral foramen magnum and originating from the left VA's dural sheath, was adjacent to the medulla and spinal cord (Figure 4).

**Figure 5:**
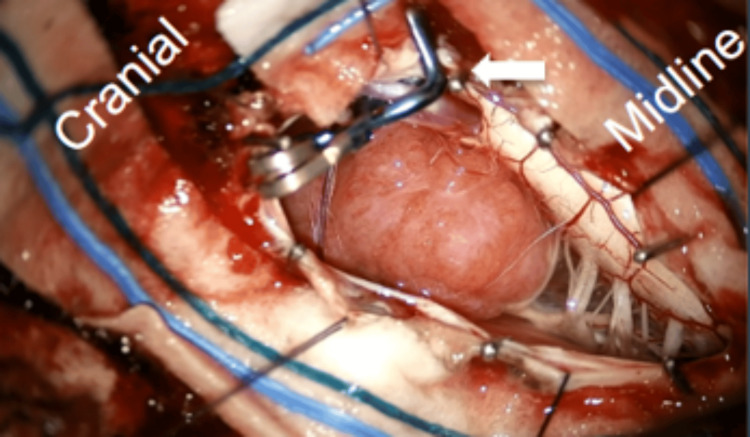
The left PICA (white arrow) was freed from the tumor and transposed away from the surgical field by using a temporary fenestrated aneurysm clip and secured to the dura edge. PICA: posterior inferior cerebellar artery.

The left PICA was observed traversing the surface of the tumor. This vessel was carefully detached from the tumor and transposed away from the surgical field using a Yasargil 5 mm curved temporary fenestrated clip, which was then secured to the edge of the dura mater (Figure 5).

**Figure 6:**
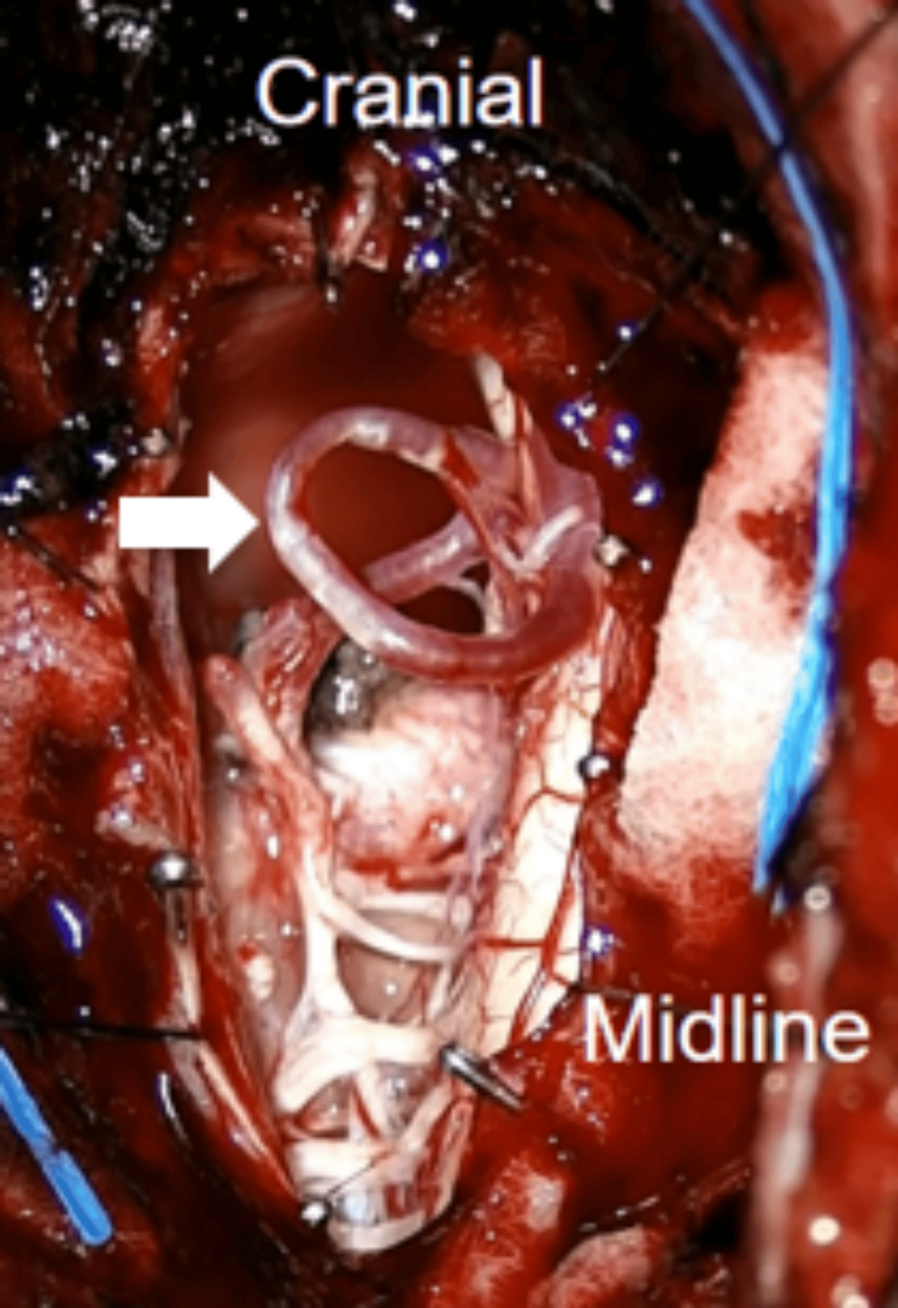
Following gross tumor resection, the fenestrated aneurysm clip was taken out, allowing the repositioning of the left PICA (white arrow) to its initial location. PICA: posterior inferior cerebellar artery.

Microsurgical techniques, including central debulking and peripheral dissection, were used to achieve gross total excision. The transposed vessel was periodically monitored using Doppler ultrasound during surgery. At the end of the surgery, the transposed vessel was released and bathed with papaverine solution. A watertight duraplasty was performed. The wound was closed in layers without postoperative complications (Figure 6).

Postoperative course

**Figure 7:**
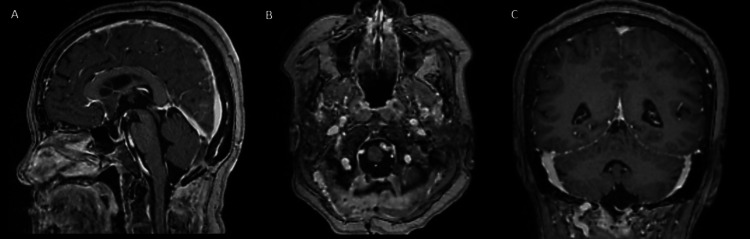
(A) Axial, (B) sagittal, and (C) coronal T1-weighted MRI with gadolinium enhancement confirming complete excision of the previously seen mass.

The patient was extubated on the day of surgery and recovered without complications, being discharged on postoperative day 3. Histopathology confirmed angiomatous meningioma, CNS WHO grade 1. A three-month postoperative MRI showed complete tumor excision with no residual tumor (Figure 7).

## Discussion

FMMs constitute a rare and complex category of intracranial tumors, primarily due to their anatomical positioning at the craniocervical junction and their close proximity to vital neurovascular elements. The foramen magnum houses several critical neuroanatomical and vascular structures. Among these neural components are the cerebellar tonsils, the inferior vermis, the fourth ventricle, the caudal medulla, the lower cranial nerves (CNs) IX to XII, the rostral segment of the spinal cord, and the upper cervical nerves (C-1 and C-2) [6].

The principal arterial structures located within the foramen magnum include the vertebral arteries, the PICAs, and the anterior and posterior spinal arteries, along with the meningeal branches that arise from the vertebral, external, and internal carotid arteries [6]. The VA passes through the foramen transversarium and ascends to the C1 vertebra, where it curves over the lateral aspect of the posterior arch. Subsequently, it moves rostrally to enter the dura mater, situated just beneath the lateral edge of the foramen magnum, near the occipital condyle. Typically, the intradural segment of the artery branches into the posterior spinal artery and the PICA; however, there are documented variations in the origin of the PICA, which may arise at, above, or below the foramen magnum [6,7].

FMMs arise from the anterior aspect of the inferior third of the clivus, extending to the superior margin of the C2 vertebral body. Laterally, these tumors grow from the jugular tubercle to the laminae of C2, whereas posteriorly, they are located between the anterior margin of the occipital squama and the spinous process of C2 [4].

Anatomical considerations

The complex anatomy of the foramen magnum requires a comprehensive understanding of the tumor's interactions with critical anatomical structures, particularly the vertebral arteries and the lower cranial nerves. The VA, which transitions from the V3 to V4 segment as it ascends through the foramen magnum, presents significant concerns due to its anatomical variations and its close proximity to FMMs. In instances where the tumor is located beneath the VA, the lower cranial nerves are generally displaced posteriorly and superiorly, thus minimizing potential complications during surgical intervention. In contrast, for meningiomas that arise above the VA, the exact positioning of the lower cranial nerves remains uncertain prior to surgery, necessitating diligent efforts to identify and safeguard these nerves throughout the operation [8,9].

Preoperative neuroimaging to delineate vascular anatomy and potential encasement is paramount in surgical planning.

Surgical challenges and technique

FMMs have the potential to infiltrate the dura mater, particularly at the junction of the V3 and V4 segments, leading to the formation of a dural ring. At this anatomical site, collagen fibers from the dura can penetrate the adventitia and media layers [10]. Surgical interventions aimed at excising the dura in this area carry a considerable risk of vascular injury. Thus, it is prudent to preserve a coagulated margin of dura around the VA to ensure safety [4]. The Bruneau and George classification system, along with the modifications introduced by Li et al., offers an extensive framework for the categorization of these tumors, taking into account their compartment of origin as well as their axial and cranio-caudal relationships to the VA [4,11]. This classification aids in surgical planning by predicting cranial nerve displacement and identifying optimal access routes.

In this case, a midline suboccipital craniotomy was selected, diverging from the standard posterolateral technique typically advised for lateral FMMs based on Bruneau’s classification. This choice was guided by the anatomical pathway established by the displacement of the neuroaxis and the location of the tumor. Furthermore, PICA transposition using a temporary fenestrated aneurysm clip was achieved, marking an innovative modification aimed at enhancing surgical access while maintaining vascular integrity, while deliberately selecting this approach over alternatives like vascular loops or BEM-sheet fixation. This approach facilitated the safe excision of the tumor without necessitating extensive bone removal, thereby minimizing operative morbidity.

Comparative techniques for PICA transposition

The mobilization of the PICA during FMM surgery is a critical maneuver, particularly when attempting to access ventral lesions via midline or paramedian approaches. Given the proximity to vital neurovascular structures, several transposition strategies have been described, each with distinct benefits and limitations [12-15].

Techniques involving a vascular loop have been well described. These methods offer flexibility and avoid the bulk of metallic instrumentation. However, they often require additional dissection space and meticulous placement, challenges in deep or narrow operative fields. Furthermore, they risk kinking the vessel and cause endothelial injury when tensioned and increase torsion hazards during dynamic retraction. 

Sling techniques using materials like BEM-sheet or Gore-Tex® provide gentle displacement and tack to adjacent structures, such as the dura or muscle, but methods may lack the stability required for prolonged or manipulative procedures, especially in midline or ventral approaches where dynamic retraction forces are involved. Moreover, this fixation limits intraoperative repositioning. 

Advantages of PICA transposition with a temporary fenestrated aneurysm clip

A temporary fenestrated aneurysm clip was used to secure the PICA atraumatically in our case. This technique offered several advantages, including stable fixation without the need for additional suturing or adhesive material, as the fenestrated clip provided consistent and firm anchorage of the artery throughout the procedure. Furthermore, the fenestrated design preserved vascular integrity by allowing the artery to be encircled gently without occlusion, thereby preserving patency and reducing the risk of vasospasm. The clip method also significantly enhanced time efficiency compared to loop-based or adhesive anchoring techniques, reducing setup time and minimizing intraoperative delay to allow greater focus on tumor resection. Additionally, the clip enabled dynamic repositioning with instantaneous release and reapplication during tumor dissection; concerns that the clip head might interfere with instruments were mitigated by strategic placement outside the working axis and by the surgical team's familiarity with clip management.

By displacing the PICA away from the operative corridor, we reduced direct manipulation of the artery, thereby minimizing risks of vasospasm, ischemia, or mechanical injury. The clip provided stable fixation and facilitated a clean operative field for tumor dissection. This approach ultimately enabled gross total resection (Simpson grade II) while preserving the integrity of vascular and neural structures.

Comparative approaches and risks

Alternative approaches, such as the extreme lateral or posterolateral approaches, frequently necessitate VA transposition to access ventral FMMs. Although these methods can be effective, they are associated with significant risks, such as potential injury to the VA, the formation of pseudoaneurysms, and the development of strictures resulting from excessive thermal coagulation [16,17]. Complications associated with the manipulation of lower cranial nerves and the destabilization of the occipital condyle present significant concerns in these surgical techniques. However, our midline approach, which incorporates PICA transposition, effectively mitigated these risks while successfully attaining a Simpson grade 2 resection.

The postoperative outcomes in this case were favorable, with gross total tumor resection confirmed intraoperatively and on follow-up imaging. The patient’s early discharge and smooth recovery underscore the efficacy of this approach. Although the midline approach combined with PICA transposition played a crucial role in addressing this particular case, its suitability for other FMMs depends upon specific tumor attributes, such as their axial and cranio-caudal orientation in relation to the VA.

## Conclusions

This case underscores the feasibility and effectiveness of PICA transposition during resection of a ventrolateral FMM. The innovative use of a fenestrated clip to secure the transposed vessel facilitated safe tumor resection while maintaining vascular integrity. This technique represents a promising strategy for addressing intricate lesions in the foramen magnum area, yielding positive clinical results. Future studies with larger cohorts and long-term follow-ups are essential to confirm the reproducibility and outcomes of this approach in the comprehensive management of FMMs.
